# Rare SMA Patients: A Comprehensive Look at Clinical Features, Genetic Profiles and Therapeutic Approaches

**DOI:** 10.3390/ijms27104301

**Published:** 2026-05-12

**Authors:** Kristina Mikhalchuk, Svetlana Artemieva, Viktoria Zabnenkova, Maria Akhkiamova, Elena Dadali, Galina Rudenskaya, Peter Sparber, Olga Rybakova, Yulia Papina, Anastasiya Monakhova, Irina Shulyakova, Dmitriy Saiko, Svetlana Zhiteneva, Alexander Polyakov, Olga Shchagina

**Affiliations:** 1 Research Centre for Medical Genetics, Moskvorechye St. 1, Moscow 115522, Russiaalbmasha@gmail.com (M.A.);; 2 Russian Children Neuromuscular Center, Veltischev Clinical Pediatric Research Institute of Pirogov Russian National Research Medical University, Taldomskaya Str. 2, Moscow 125412, Russia; artemievasb@mail.ru (S.A.);; 3 Republican Clinical Hospital named after M.I. Kalinin, Ilyich Av. 14, Donetsk 283003, Russia; 4 State Budgetary Healthcare Institution of the Moscow Region “Lukhovitskaya Hospital”, Lukhovitsy, Mira Str. 39/5, Moscow 140500, Russia

**Keywords:** novel variant, subtle variant, intragenic variant, compound heterozygous, gene therapy, spinal muscular atrophy, 5q SMA, *SMN1*

## Abstract

Spinal muscular atrophy 5q (5q SMA) is one of the most prevalent autosomal recessive disorders globally. The underlying cause of 5q SMA is attributed to variants in *SMN1*. To date, there are no reported cases of gene-based therapy in rare patients with 5q SMA caused by subtle *SMN1* variants of unknown clinical significance. We included 10 patients with the clinical manifestations of 5q SMA associated with intragenic variants in combination with a heterozygous *SMN1* deletion in this retrospective study. Previously reported pathogenic or likely pathogenic variants were identified (e.g., c.*3+1del, c.815A>G (p.Tyr272Cys), and c.821C>T (p.Thr274Ile)). Variants of unknown clinical significance were also found, including a recurrent, previously unreported variant c.80A>C (p.Gln27Pro). We also report detailed molecular genetic and clinical data on 9 patients with 5q SMA. In addition, we provide results from the cohort of patients with gene-based therapy, consistent with data from patients with a homozygous *SMN1* deletion.

## 1. Introduction

The most common form of SMA is proximal SMA associated with *SMN1* (5q spinal muscular atrophy; 5q SMA), which accounts for approximately 85% of all cases in this group [1]. In the Russian Federation, based on data from three pilot projects on neonatal screening for 5q SMA, the frequency of the disease ranges from 1 in 7801 to 1 in 9035 newborns [2,3], whilst the carrier frequency of the deletion is 1 in 38 people [4]. The estimated carrier frequency of 5q SMA ranges from 1 in 25 to 1 in 50 in different ethnic groups [5,6]. 

The clinical presentation of 5q SMA is characterized by progressive flaccid paralysis caused by degeneration of α-motor neurons in the anterior horns of the spinal cord. Five clinical subtypes of 5q SMA are differentiated based on age of onset, disease severity and life expectancy [7]. These subtypes represent allelic variations caused by pathogenic *SMN1* variants in a homozygous or compound heterozygous state.

The most common genetic mechanism underlying 5q SMA is associated with large deletions or gene conversion leading to the absence of exon 7 of *SMN1*. These are observed in 95–98% of patients in a homozygous state [8]. A less common type of variant is associated with intragenic pathogenic variants, which are commonly found in patients with compound heterozygosity, including deletion of one *SMN1* allele. The proportion of patients with such variants is estimated by various researchers to range from 1.8% to 4.8% of all 5q SMA cases [8,9,10,11].

There are three FDA-approved (Food and Drug Administration, Silver Spring, MD, USA) medications available for gene-based therapy of 5q SMA: Onasemnogene abeparvovec-xioi (marketed as Zolgensma^®^ (NOVARTIS PHARMA, AG, Basel, Switzerland)), Nusinersen (marketed as Spinraza^®^ (BIOGEN NETHERLANDS, B.V., Badhoevedorp, The Netherlands)) and Risdiplam (marketed as Evrysdi^®^ (F.Hoffmann-La Roche, Ltd., Basel, Switzerland)) [12]. These medications are available to patients with 5q SMA in Russia, with expenses covered by the state-funded ‘Circle of Kindness’ program.

A limited number of cases reported in global literature describe patients with 5q SMA caused by a heterozygous deletion of *SMN1* in combination with a subtle variant in a compound heterozygous state, accompanied by detailed clinical presentations. Identifying the pathogenicity of novel missense variants remains challenging. Additionally, it is frequently challenging to definitively verify the subtle variant in the sequence of *SMN1*. The value of RNA analysis is also a point of discussion [13], considering the high homology of nucleotide sequences between *SMN1* and *SMN2*, which complicates the assignment of transcripts to a specific gene. These diagnostic challenges may reduce the accuracy of identifying 5q SMA, potentially delaying the initiation of gene-based therapy. Moreover, current global literature has no reports on results of gene-based therapy for patients with 5q SMA caused by intragenic variants of uncertain clinical significance. Data on long-term outcomes in such cases are limited compared with cases with the homozygous deletion of exon 7 of *SMN1*. This study reports the results of molecular genetic diagnosis in patients with suspected 5q SMA caused by heterozygous deletion of *SMN1* in combination with intragenic variants, including detailed descriptions of disease progression and treatment outcomes.

## 2. Results

This retrospective study included 10 patients with clinical 5q SMA features, associated with an intragenic variant in combination with a heterozygous *SMN1* deletion. This study included six patients from our previous research (DNA# 6766.1, 8118.1, 8826.1, 9804.1, 9868.1, 10976.1) [9], for whom clinical examination data were available following the initiation of gene-based therapy (Tables S1 and S2). Additionally, between 2023 and 2025, four patients with suspected 5q SMA and a heterozygous deletion of exon 7 of *SMN1* were identified at the Research Centre for Medical Genetics (Table 1). Thus, clinical examination data following the initiation of gene-based therapy were available for nine patients. Molecular genetic testing of these four patients identified intragenic variants in *SMN1/SMN2* in combination with the heterozygous deletion of exon 7 of *SMN1*.

Then, we analyzed the identified intragenic variants, along with the clinical data of patients who were referred to the Research Centre for Medical Genetics for molecular genetic testing.

Between 2023 and 2025, molecular genetic testing of patients identified the previously described variants c.*3+1del and c.821C>T (p.Thr274Ile), the pathogenicity and causality of which are proven. A novel variant of uncertain clinical significance, c.80A>C (p.Gln27Pro), was identified in two unrelated probands with suspected 5q SMA.

### 2.1. Analysis of the Identified Variant of Unknown Clinical Significance in SMN1/SMN2

A patient, 8080.1, with suspected 5q SMA (clinical description of patient 8080.1 is provided in Table S1) has a heterozygous deletion of exons 7 and 8 of *SMN1* and a homozygous deletion of exons 7 and 8 of *SMN2* by Multiplex Ligation-dependent Probe Amplification (MLPA) analysis. Consistently, direct automated Sanger sequencing identified a variant of uncertain clinical significance in heterozygous state at exon 1 of *SMN1/SMN2* — c.80A>C (p.Gln27Pro). The c.80A>C (p.Gln27Pro) variant is not present in the gnomAD v4.1.0 population frequency database. The c.80A>C (p.Gln27Pro) variant affects a moderately conservative amino acid. The results of in silico variant effect prediction algorithms indicate a pathogenic (Revel, FATHMM, DANN) effect of this variant on protein structure; however, according to the AlphaMissense prediction, the effect of this variant on protein structure is unknown (0.435).

**Figure 1:**
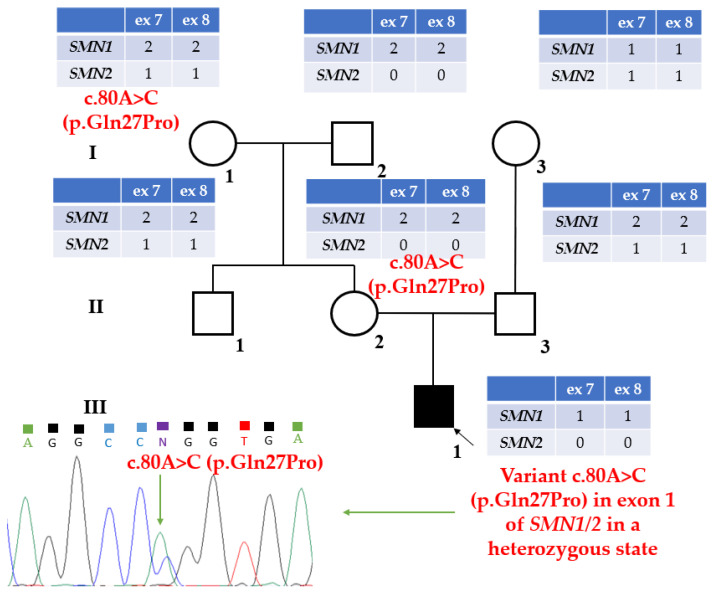
Molecular genetic data for three generations of patient 8080.1’s family. The tables show the copy numbers of exons 7 and 8 of *SMN1* and *SMN2* as determined by MLPA. The chromatogram indicates that the variant in exon 1 of *SMN1/2* is in a heterozygous state.

As the homozygous deletion of exons 7 and 8 of *SMN2* was identified in this patient and the variant c.80A>C (p.Gln27Pro) was identified in the heterozygous state, a complex rearrangement at the SMN locus was suggested, as a result of which the variant was identified in a heterozygous state (Figure 1). All available family members of the patient 8080.1 were tested for the presence of the c.80A>C (p.Gln27Pro) variant by direct automatic Sanger sequencing. This variant was identified in the mother (II.2) and maternal grandmother (I.1).

To study the presence of a complex rearrangement, available family members of the patient from two generations were genotyped. The number of copies of exons 1–6 of *SMN1/SMN2* was determined by MLPA. One copy of exons 1–6 of *SMN1/SMN2* was identified, inherited by patient (III.1) from mother (II.2), who in turn inherited this copy from her father (I.2) (Figure 1). Since the c.80A>C (p.Gln27Pro) variant was inherited by patient (III.1) from his mother (II.2) and maternal grandmother (I.1), and a single copy of exons 1–6 of *SMN1/SMN2* from his mother (II.2) and maternal grandfather (I.2), a recombination was assumed to have occurred during the patient’s mother’s meiosis.

**Figure 2:**
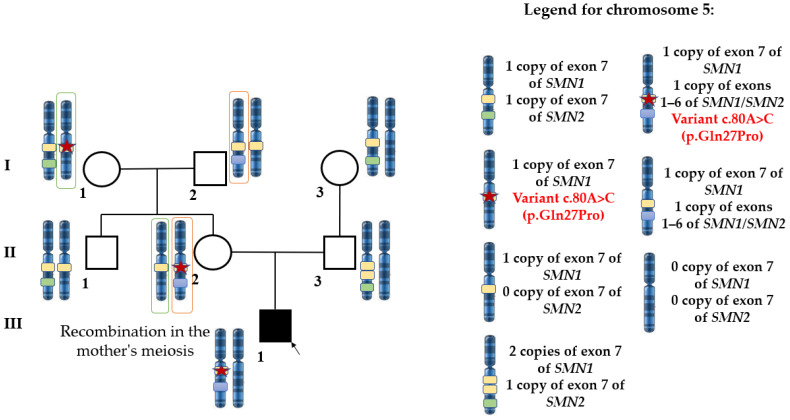
Allele distribution for variants in *SMN1* and *SMN2* in the family of patient 8080.1.

Since the genotypes of the father (II.3) and the paternal grandmother (I.3) are known, it was assumed that the father (II.3) carries an *SMN1* duplication on one allele and an *SMN1* deletion on the other (genotype ‘2+0’) (Figure 2). This parent was also analyzed for carriage of known *SMN1* duplication markers: variants c.*3+80T>G (g.27134T>G) (rs143838139) in intron 7 and c.*211_*212del (g.27706_27707delAT) in exon 8 of *SMN1* (NM_000344.3). These variants were not detected.

In patient 6.3472 with suspected 5q SMA, variant c.80A>C (p.Gln27Pro) was also detected in combination with a heterozygous deletion of exons 7–8 of the *SMN1* gene. The parents were not available for segregation analysis. 

### 2.2. Clinical Outcome of the Disease in Patients with Intragenic Variants and Results of Gene-Based Therapy

Subsequently, we analyzed clinical data from four patients (DNA# OD1, 8080.1, 6.3472, 6.4308) with suspected 5q SMA who were referred to the Research Centre for Medical Genetics between 2023 and 2025. These cases were evaluated alongside six patients included in our previous study (DNA# 6766.1, 8118.1, 8826.1, 9804.1, 9868.1, 10976.1) [9] for whom clinical data were available following gene-based therapy. There are no pathognomonic symptoms specific to 5q SMA. All patients had muscle hypotonia, reduced or absent tendon reflexes, reduced muscle strength, limited joint mobility in the limbs and fasciculations of the tongue. These findings supported a clinical diagnosis of 5q SMA. All patients with intragenic variants in combination with the heterozygous deletion of exon 7 of *SMN1* had a clinical phenotype of the classic 5q SMA types, indistinguishable from the phenotype of patients with a homozygous deletion of exon 7 of *SMN1* (Tables S1 and S2).

Molecular genetic testing not only allows us to confirm the diagnosis in a patient with 5q SMA, but also to analyze the carrier status of pathogenic variants in *SMN1* in the patient’s family, in addition to determining the appropriate gene-based therapy. We had data from clinical examinations of patients with 5q SMA and variants in *SMN1/SMN2* in combination with a heterozygous deletion of *SMN1* following the initiation of gene-based therapy (Table 2). Patient 8080.1 had a homozygous *SMN2* deletion, which made it impossible to assign him existing gene-based medications.

Nusinersen was assigned to six patients; two of these were subsequently injected with Onasemnogene abeparvovec after starting Nusinersen treatment. Risdiplam was assigned to three patients. All medications were administered in accordance with the manufacturers’ protocols. Motor development was assessed during clinical examinations by neurologists, using the HFMSE, RULM and CHOP INTEND scales. Informed consent forms were signed with patients or legal representatives before the start of therapy. Patients or legal representatives were informed about the mechanism of action of medications, potential side effects of medications and the lumbar puncture procedure. The age range at the start of therapy with Nusinersen was 6 months to 17 years, with Risdiplam 17 to 47 years, and with Onasemnogene abeparvovec 12 months to 15 months (following the initiation of Nusinersen therapy at 6 months in both patients). Data on motor development assessment using the HFMSE and RULM scales were available for only four patients (Table 2) with 5q SMA type III.

Baseline assessment (before the first introduction of the pathogenetic medication) on the HFMSE and RULM scales varied significantly among patients due to the heterogeneity of the cohort. Only two patients, 9868.1 and 6766.1, showed significant positive improvement on the HFMSE scale—9 and 10 points, respectively—indicating the acquisition of new motor skills, while the conditions of the others were assessed as stable. According to treating physicians and relatives caring for the patients, the patients demonstrated a positive response to the therapy, which is a subjective assessment. However, these results are also observed in patients with a homozygous *SMN1* deletions and 5q SMA type III.

To compare data on the HFMSE and RULM scales in the group of patients with intragenic variants in *SMN1* in combination with a heterozygous deletion of exon 7 of *SMN1*, we analyzed data on the HFMSE and RULM scales in the group of patients with the homozygous *SMN1* deletion with 5q SMA type III (Table 3). We used no statistical analysis of the results due to the limited number of patients in the comparison groups. In the group of patients with the homozygous *SMN1* deletion (n = 26) who were assigned Nusinersen, the median age at the start of gene-based therapy was 9 years and 4 months; this group also included 22 patients with three copies of *SMN2* and four patients with four copies of *SMN2*. In the group of patients with the homozygous *SMN1* deletion (n = 2) who were assigned Risdiplam, the median age at the start of gene-based therapy was 9 years and 3 months; two patients were identified to carry three copies of *SMN2*. In the group of patients with intragenic variants in *SMN1* in combination with the heterozygous deletion of exon 7 of *SMN1*, as in the group of patients with the homozygous *SMN1* deletion, there were patients who demonstrated either gain or loss of points on the HFMSE and RULM scales, as well as the same scores 1–3 years after the start of gene-based therapy.

## 3. Discussion

To confirm the pathogenicity of intragenic variants in *SMN1*, current criteria require that either: (1) the variant has been previously described in peer-reviewed studies by other researchers; or (2) the *SMN1* gene product or its subclone has been sequenced using long-range PCR [7]. The application of functional analysis (e.g., PS3 or BS3 criteria of ACMG) remains debated [13], primarily due to the high sequence homology between *SMN1* and *SMN2*, which complicates the attribution of transcripts to a specific gene. However, the identification of a variant of uncertain clinical significance located specifically in *SMN1* —and, as a result, the application of the PM3 criterion which assumes the presence of a variant in a trans-configuration with a known pathogenic variant in recessive disorders—is not always sufficient to classify a variant of uncertain clinical significance as likely pathogenic. In clinical practice, the diagnosis of 5q SMA in patients with a heterozygous *SMN1* deletion and a variant of unknown clinical significance is based on segregation analysis and the patient’s clinical symptoms matching those of 5q SMA.

In this study, the variant of unknown clinical significance, c.80A>C (p.Gln27Pro), in combination with a heterozygous deletion of exon 7 of *SMN1* was identified in two patients with suspected 5q SMA (6.3472 and 8080.1). The attending physician initiated gene-based therapy for patient 6.3472, based on a neurological examination and instrumental methods (electroneuromyography (ENMG)). An improvement in motor skills was subsequently observed (Table S2). Patient 8080.1 had a homozygous *SMN2* deletion, which made it impossible to assign him existing gene-based medications. Thus, the clinical diagnosis of 5q SMA type III in patient 8080.1 was based on clinical data and the presence of the intragenic variant in combination with the heterozygous *SMN1* deletion.

We suggest the clinical diagnosis of 5q SMA type III with the ability to walk without assistance in patient 8080.1, who carries 0 copies of *SMN2*, as well as in patient 6.3472, with disease manifestation at the age of 18, is associated with the fact that the c.80A>C (p.Gln27Pro) variant may cause a slight reduction in the affinity of SMN for Gemin2. Thus, the data in the literature suggest that the development of the 5q SMA phenotype may be caused by defects in snRNP assembly, which result from reduced SMN expression. The assembly of Sm protein heptameric rings on snRNA (Sm-nuclei), which are essential for snRNP function, is mediated by the SMN complex. The specific assembly of the Sm-nucleus depends on the recognition of Sm proteins and snRNA by subunits containing SMN/Gemin2 and Gemin5, respectively. Small nuclear ribonucleoprotein particles (snRNPs) represent the major class of non-coding RNA-protein complexes that perform critical roles in post-transcriptional gene expression, including pre-mRNA splicing and the suppression of premature termination. The SMN complex consists of SMN, Gemins 2–8 and Unrip. Sm proteins are recognized by a subunit comprising SMN and Gemin2. Sequence alignments of SMN indicate that most of the residues involved in the interaction with Gemin2 are conserved [17,18]. The SMN fragment containing amino acid residues 1–62 (exons 1–2b) binds to Gemin2 in the same way as fragments containing residues 1–209 (exons 1–4) and full-length SMN. The complex formed between SMN26–51 and Gemin295–280 (24 kDa) was shown to be highly soluble and monodisperse in solution, with no signs of oligomerization [18]. Ogawa et al. reported that the affinity of Gemin2 for truncated SMN containing exons 1–5 is reduced by the missense variant c.131A>T (p.Asp44Val), both in mammalian studies and in vitro experiments [19].

The detection of a variant of unknown clinical significance according to ACMG criteria in patients with suspected 5q SMA highlights the complexity of molecular genetic diagnosis of 5q SMA, since only patients with biallelic pathogenic and likely pathogenic variants are considered for initiation of gene-based therapy. Currently, physicians in clinical practice focus on the combination of the patient’s clinical data and the results of molecular genetic testing. In patient 8118.1 [11], who carried a heterozygous *SMN1* deletion and a variant of unknown clinical significance (c.835-18_835-15delCCTT) in *SMN1*, clinical improvement in motor function was observed during gene-based therapy, evidenced by a four-point increase on the RULM functional scale, which is considered clinically significant. Thus, the diagnosis of 5q SMA type III in patient 8118.1 s highly probable.

Clinicians should remain vigilant for the clinical manifestations of 5q SMA, despite the absence of a highly specific phenotype and pathognomonic clinical features of the disease. The investigation of the genetic mechanisms of 5q SMA is currently a significant task in medical genetics, particularly considering the availability of disease-modifying therapy for 5q SMA. The ability to identify the genetic basis of 5q SMA is critically important for patients, since only patients with biallelic pathogenic or likely pathogenic variants in *SMN1* are eligible for gene-based therapy [20]. At present, medicinal therapy for 5q SMA includes medications for pathogenetic (replenishment of SMN protein deficiency) and symptomatic treatment. It is recommended to start gene-based therapy for 5q SMA as early as possible in all patients with a confirmed genetic diagnosis (regardless of age at disease onset, current age and baseline functional status) in the aim of achieving the best therapeutic effect [21].

The cohort of patients in this study who received gene-based therapy was heterogeneous and included patients with various clinical subtypes of the disease. The general conclusion, consistent with the results of randomized clinical trials of these medications, is that the optimal response is obtained with the earliest possible initiation of therapy and the lowest baseline disease severity. Only two patients demonstrated an improvement in motor skills on the HFMSE scale. The general impressions of the treating physicians, and also of the relatives caring for the patients, were positive. A number of changes were noted that significantly affect the quality of life of patients and their families, but which are not reflected in formal motor development scales. Thus, long-term curation is required to assess the response in more severely affected patients in actual clinical practice. This conclusion is consistent with available data on long-term curation during Nusinersen therapy in clinical trials [22].

Despite the fact that intragenic variants are an extremely rare genetic cause of 5q SMA, with limited experience of gene-based therapy in this patient group, such therapy can be administered to a patient if a physician is confident of the diagnosis of 5q SMA.

## 4. Materials and Methods

### 4.1. Patient Recruitment

This retrospective study included 10 patients with clinical 5q SMA features, associated with an intragenic variant in combination with a heterozygous *SMN1* deletion. The inclusion criteria were: (1) a clinical diagnosis of 5q SMA, (2) the intragenic variant detected in combination with the heterozygous *SMN1* deletion, (3) administration of gene-based therapy and (4) available neurological examinations conducted by physicians before and after therapy initiation. This study also included a patient, 8080.1, with clinical manifestations of 5q SMA and a homozygous *SMN2* deletion, who was not a candidate for gene-based therapy. However, the same variant of unknown clinical significance was identified in this patient as in patient 6.3472, who was assigned gene-based therapy.

This research included DNA samples from five unrelated patients referred to the Research Centre for Medical Genetics for testing for 5q SMA between 2023 and 2025. Available DNA samples from relatives of patient 8080.1 were also analyzed to identify the origin of the allele. Six patients with 5q SMA caused by a heterozygous deletion of exon 7 of *SMN1* and an intragenic variant in *SMN*, with available clinical data and results of clinical examination following gene-based therapy (#DNA: 9868.1, 6766.1, 10976.1, 9804.1, 8826.1, 8118.1) have been reported by us previously [9].

Informed consent for molecular testing and publication of the results of research was obtained from all patients or their legal representatives. The research was approved by the Ethics Committee of the Research Centre for Medical Genetics, Moscow, Russia (approval number 11/1, dated 23 November 2021). Informed consent was obtained from patients or their legal representatives before the initiation of therapy. Patients or their legal representatives were informed about the pharmacological mechanism of action of the medications, potential side effects and the procedure for lumbar puncture. The study was conducted in accordance with the Declaration of Helsinki.

### 4.2. Molecular Genetic Diagnostic Methods Used in the Study

DNA was extracted from peripheral blood leukocytes using the Wizard^®^ Genomic DNA Purification Kit (Promega, Madison, WI, USA) in accordance with the manufacturer’s protocol. The copy number of *SMN1* and *SMN2* gene exons was determined by MLPA using the SALSA MLPA Probemix P060 SMA Carrier Kit (MRC Holland, Amsterdam, The Netherlands) and SALSA MLPA Probemix P021 SMA (MRC Holland, Amsterdam, The Netherlands), in accordance with the manufacturer’s protocol. The reaction product was detected by fragment analysis on an ABI Prism 3500 device (Applied Biosystems, Thermo Fisher Scientific, Waltham, MA, USA).

In total, 10 unrelated probands with a heterozygous deletion of exon 7 of *SMN1* were included in this study. The coding and adjacent non-coding regions of exons 1–8 of *SMN1/SMN2* were sequenced. Variants in *SMN*/*SMN2* were identified using automated Sanger sequencing with both forward and reverse primers on an ABI Prism 3500 (Applied Biosystems) device, in accordance with the manufacturer’s protocol. The template for sequencing comprised DNA fragments obtained following PCR amplification with specific oligonucleotide primers [23].

The high homology between *SMN1* and *SMN2* presents a methodological limitation: it is challenging to ascertain whether the identified intragenic subtle variants originate from *SMN1* or *SMN2*. *SMN1* is located in the repeat region of chromosome 5 at locus 5q12.2–q13.3 in combination with *SMN2*. Although *SMN2* has significant similarities to *SMN1*, it is differentiated by a single nucleotide difference in the coding sequence. Specifically, in exon 7, the single nucleotide variant (SNV) c.840C is unique to *SMN1*, whereas c.840T is unique to *SMN2* (p.Phe240=) (rs4916) (ENST00000380707; NM_000344.4). Different genetic variations between these two genes play a crucial role in the pathogenesis of 5q SMA, emphasizing the importance of understanding these differences for genetic testing and therapeutic strategies.

The reference transcript selected for this study was NM_000344.4 (NP_000335.1). Variants were classified based on the criteria established by the American College of Medical Genetics and Genomics in 2015 [13]. Various pathogenicity prediction tools were employed for assessing the impact of missense nucleotide sequence variants: MT, FATHMM, DANN, MetaLR, fitCons, AlphaMissense, PrimateAI, GERP, GenoCanyon, Revel, BayesDel and SIFT.

In search of identified *SMN1* duplication markers (variants NM_000344.4:c.*3+80T>G (g.27134T>G) (rs143838139) and c.*211_*212del (g.27706_27707delAT)) in the father 8080.3 (II.3) of patient 8080.1, allele-specific ligation was performed, followed by visualization of the reaction results on a polyacrylamide gel using probes for the detection of *SMN1* duplication genetic markers [4].

## 5. Conclusions

Global literature reports a limited number of cases of patients with 5q SMA caused by a heterozygous *SMN1* deletion in combination with an intragenic variant. In large cohort studies, intragenic variants of unknown clinical significance have identified, which, due to limitations, cannot be reclassified as pathogenic or likely benign/benign. However, based on the clinical manifestations of 5q SMA in a patient with an identified intragenic variant in combination with a heterozygous *SMN1* deletion, physicians may initiate gene-based therapy for patients. Furthermore, to date, no clinical data are available on such rare patients with 5q SMA treated with FDA-approved gene-based therapy. We report, for the first time, the results of clinical examinations of available rare patients with 5q SMA caused by a heterozygous *SMN1* deletion in combination with an intragenic variant, after the administration of gene-based therapy. We also report a variant of unknown clinical significance (c.80A>C (p.Gln27Pro)) identified in two unrelated patients in our cohort who presented with clinical features of 5q SMA. In the group of patients with intragenic variants in *SMN1* in combination with the heterozygous deletion of exon 7 of *SMN1*, as in the group of patients with the homozygous *SMN1* deletion, there were patients who demonstrated either gain or loss of points of motor development scores, as well as the same scores 1–3 years after the start of gene-based therapy. However, long-term curation is required in clinical practice to evaluate the treatment response in patients with 5q SMA associated with intragenic variants.

## Figures and Tables

**Table 1:** Genotypes of four patients with suspected 5q SMA, assessment of the pathogenicity of the identified variants and the origin of the variant allele in the available family.

№	DNA	Copy of *SMN1*	Copy of *SMN2*	Variant NM_000344.4 (NP_000335.1)	Pathogenicity (ACMG Criteria or Studies)	Parental Origin of Variants	5q SMA Type
1	8080.1	1	0	c.80A>C (p.Gln27Pro)	VUS (PM2, PM3, PP3)	Maternal	III
2	6.3472	1	1	c.80A>C (p.Gln27Pro)	VUS (PM2, PP3)	No data	III
3	6.4308	1	2	c.*3+1del	Pathogenic [14]	No data	III
4	OD1	1	1	c.821C>T (p.Thr274Ile)	Pathogenic [15,16]	No data	I

**Table 2:** Clinical data for patients (n = 9) with variants in *SMN1/SMN2* in combination with a heterozygous *SMN1* deletion who were assigned gene-based therapy, and an evaluation of motor development in dynamics.

DNA	*SMN1* Variants (NM_000344.4)	5q SMA type	Onset of the Disease	Initiation of Gene-Based Therapy	Initial Data on Motor Scales	Dynamics at the Time of the Last Introduction
9868.1	c.13del (p.(Ser5Alafs*35))	III	4 years	Nusinersen (6 years)	HFMSE – 46 (6 years)	HFMSE – 55 (7 years) HFMSE – 52 (9 years)
10976.1	c.815A>G (p.Tyr272Cys)	I	10 months	Nusinersen (1 year), Onasemnogene abeparvovec (1 year and 3 months)	No data	No data
6766.1	c.815A>G (p.Tyr272Cys)	III	1 year and 5 months	Nusinersen (14 years)	HFMSE – 8 RULM – 24 (14 years)	HFMSE – 18 RULM – 22 (17 years)
9804.1	c.821C>T (p.Thr274Ile)	III	2.5 years	Nusinersen (16 years)	HFMSE – 21 (16 years)	HFMSE – 21 (17 years)
8826.1	c.821C>T (p.Thr274Ile)	III	10 months	Nusinersen (7 years)	No data	CHOP INTEND – 41 (9 years)
8118.1	c.835-18_835-15del	III	15 years and 4 months	Risdiplam (17 years)	HFMSE – 20 RULM – 26 (17 years)	HFMSE – 20 RULM – 30 (18 years)
OD1	c.821C>T (p.Thr274Ile)	I	3.5 months	Nusinersen (6 months), Onasemnogene abeparvovec (1 year)	No data	No data
6.3472	c.80A>C (p.Gln27Pro)	III	18 years	Risdiplam (43 years)	No data	No data
6.4308	c.*3+1del	III	2 years	Risdiplam (47 years)	No data	No data

CHOP INTEND—Children’s Hospital Of Philadelphia Infant Test Of Neuromuscular Disorders; HFMSE—The Hammersmith Functional Motor Scale Expanded; RULM—Revised Upper Limb Module for 5q SMA.

**Table 3:** Analysis of data on the assessment of motor development dynamics in two groups of patients with 5q SMA.

Characteristics of Motor Development Scores	Patients with Homozygous *SMN1* Deletion	Patients with an Intragenic Variant in *SMN1*
**Nusinersen**		
Median HFMSE score at the start of therapy	37.5 points (n = 26)	21 points (n = 3)
Median HFMSE score after 1 year of therapy	47.5 points (n = 24)	38 points (n = 2)
Median HFMSE score after 3 years of therapy	29 points (n = 9)	35 points (n = 2)
Median change in HFMSE scores after 1 year of therapy	1 point (n = 24)	4 points (n = 2)
Median change in HFMSE scores after 3 years of therapy	3 points (n = 9)	7.5 points (n = 2)
Median RULM score at the start of therapy	23 points (n = 7)	24 points (n = 1)
Median RULM score after 3 years of therapy	23 points (n = 7)	22 points (n = 1)
Median change in RULM scores after 3 years of therapy	Loss of 1 point (n = 7)	Loss of 2 points (n = 1)
**Risdiplam**		
Median HFMSE score at the start of therapy	54 points (n = 2)	20 points (n = 1)
Median HFMSE score after 1 year of therapy	49 points (n = 2)	21 points (n = 1)
Median change in HFMSE scores after 1 year of therapy	Loss of 5 points (n = 2)	0 points (n = 1)

n = number of patients with available data on the HFMSE and RULM scales.

## Data Availability

The original contributions presented in this study are included in the article/Supplementary Material. Further inquiries can be directed to the corresponding author.

## Supplementary Materials

The following supporting information can be downloaded at: https://www.mdpi.com/article/10.3390/ijms27104301/s1, Reference [9] is cited in the Supplementary Materials.
