# Supplementary material for: Rare SMA Patients: A Comprehensive Look at Clinical Features, Genetic Profiles and Therapeutic Approaches
Source: Int J Mol Sci. 2026 May 12;27(10):4301. doi: 10.3390/ijms27104301 (PMC13207005; doi:10.3390/ijms27104301)
Supplement: Supplementary file 1 [file ijms-27-04301-s001.zip › ijms-4255812-supplementary.pdf]

Table S1. Clinical and instrumental data in patients with 5q SMA caused by heterozygous deletion of exon 7 of *SMN1* in combination with a previously unreported intragenic variant.

# DNA	Genotype	Early development and medical history	Clinical symptoms of the disease	Instrumental examinations
**8080.1**	*SMN1* (NM_000344.4): c.80A>C (p.Gln27Pro)<br>*SMN1* – 1 copy<br>*SMN2* – 0 copy	Early motor development was age-appropriate: he was able to hold his head up by 2 months and sit up by 6 months. The onset of the disease occurred at 8 months, when a delay in motor development became apparent.	At age 2: intelligence is slightly reduced; speech consists of single words and syllables, with impaired pronunciation; fasciculations in the tongue muscles; terminal atrophy of the tongue; chewing is weakened; phonation is reduced; holds head upright, lifts it while lying down; does not sit up independently; when seated, sits steadily with kyphosis of the back; "free shoulders" sign is present; does not stand independently; no support on feet; turns to both sides; does not crawl, does not get up on all hands and knees, maintains a knee-elbow position; diffuse hypotonia in the legs; tendinous reflexes in the hands, knees, and Achilles tendons are not evoked; movement in the joints of the upper limb is slightly limited due to muscle weakness, more so in the proximal segments; grasping of light objects with the hands is normal; muscle strength is 3–4 points; active movements in the joints of the lower limb are limited due to muscle weakness, more so in the proximal segments; muscle strength is 2–3 points.<br>At age 10: speech consists of simple phrases; facial phenotype without signs of dysmorphogenesis; fasciculations of the tongue; does not walk independently; marked muscle weakness, muscle hypotrophy, hypotonia; contractures of the wrist, interphalangeal, and ankle joints; tendon reflexes in the arms and legs are not evoked.	MRI of the muscles at age 2: diffuse involvement of the thigh and lower leg muscles is noted.<br>ENMG and EMG at age 2: the results indicate marked axonal involvement.

Table S2. Clinical data from patients with 5q SMA caused by heterozygous deletion of exon 7 of *SMN1* in combination with previously reported/unreported intragenic variants who were assigned gene-based therapy.

# DNA	Genotype	Symptoms on the first visit	Gene-based therapy (age at onset)	Symptoms (age at last examination)	Assessment of motor development scales
**9868.1 [9]**	*SMN1* (NM_000344.4): c.13del (p.(Ser5Alafs*35)) *SMN1* – 1 copy *SMN2* – 3 copies	At age 4: the chest is flattened in the anteroposterior direction; "winged scapulae"; hyperlordosis of the lumbar spine when standing upright; X-shaped leg deformity, flat-valgus foot deformity; stiffness in the ankle joints; fibrillations on the tongue; passive movements are not restricted; active movements are difficult; muscle strength is reduced; muscle tone is reduced; tendon reflexes are absent; head control is good; turns to the right and left with difficulty; sits down independently; myopathic gait; cannot perform heel-to-toe or toe-to-heel walking tests; cannot hop; does not run; climbs stairs with marked difficulty; Gowers' test is positive; posture is weakened; muscle strength in the upper and lower	Nusinersen (6 years)	At 9 years old: height 143 cm, weight 36 kg; head turns in full, shoulder lifting is not difficult; marginal tongue twitches are detected; head holds well, raises lying down; sits with kyphosis of the back, sits independently with myopathic techniques; myopathic gait with swinging to the sides; flat foot deformity; passive movements during all joints of the upper extremities are fully functional; the strength of the muscles of the upper extremities is reduced to 3 points, raises the arms above the horizontal level, tendon reflexes are not triggered; passive movements are limited in the ankle joints; the strength of the muscles of the lower extremities is reduced to 3 points	The HFMSE score at 6 years of age (after 4 injections) is 46 points, 10 meters walking is 15.38 seconds. At age 7: HFMSE - 55 points. The 6-minute test is 164 meters. At the age of 9: HFMSE - 52 points. The 6-

# DNA	Genotype	Symptoms on the first visit	Gene-based therapy (age at onset)	Symptoms (age at last examination)	Assessment of motor development scales
		extremities is reduced to 3 points, worse proximally; muscle tone in the upper and lower extremities is reduced; tendon reflexes in the upper and lower extremities are not elicited; range of motion in the hip and knee joints is full, in the ankle joints—limited.		proximally, 4 points distally; tendon reflexes are not triggered; tremor in the hands.	minute test is 126 meters.
**6766.1 [9]**	*SMN1* (NM_000344.4): c.815A>G (p.Tyr272Cys) *SMN1* – 1 copy *SMN2* – 3 copies	From the age of 1.5, parents began to notice that the patient was walking badly, often falling. The patient lost the ability to walk independently at the age of 3.	Nusinersen (14 years)	At the age of 17, he was 165 cm tall and weighed 70 kg. He had flaccid tetraparesis, hyperkyphosis, S-shaped scoliosis of the thoracolumbar region, contractures of the hip and knee joints, good head control, and the ability to sit up and walk. However, he could only turn from back to side using his hands. He also had flat-valgus feet, contractures in the knee and ankle joints, and full passive movements in all joints of the upper extremities, but limited active movements due to muscle weakness. The	At the age of 14: on the HFMSE scale - 8 points, RULM - 24 points. At the age of 17: HFMSE - 18 points, RULM - 22 points.

# DNA	Genotype	Symptoms on the first visit	Gene-based therapy (age at onset)	Symptoms (age at last examination)	Assessment of motor development scales
				strength of the muscles in the upper extremities was reduced to 3 points proximally and 3 points distally, and tendon reflexes were not elicited. Passive movements in the lower extremities were limited due to pain in the right hip joint and contractures in the knee and ankle joints. The strength of the muscles in the lower extremities was reduced to 2 points, and tendon reflexes were not elicited.	
**10976.1 [9]**	*SMN1* (NM_000344.4): c.815A>G (p.Tyr272Cys) *SMN1* – 1 copy *SMN2* – 2 copies	Examination at 10 months: malnutrition, the child's general condition is severe due to diffuse muscle hypotonia, trophic disorders, delayed physical development, passive body position, and pseudo-bulbar syndrome; the child's body is passive, hand movements are preserved, and spontaneous motor activity is reduced; respiratory failure of grade 1; ankle	At the age of 12 months, the first dose of Nusinersen was administered. At the age of 1 year and 3 months, Onasemnogene abeparvovec was	Examination data at the age of 1 year and 10 months: barrel chest deformity, limited mobility in the knee joints, equinovarus alignment and ankle joint mobility on the right, flat-valgus alignment on the left, and paresis of the arms; the patient holds their head in a sitting position, gets tired quickly, turns to both sides, does not turn	No data

# DNA	Genotype	Symptoms on the first visit	Gene-based therapy (age at onset)	Symptoms (age at last examination)	Assessment of motor development scales
		contractures; diffuse muscle hypotonia; dolichocephalic skull; weak visual fixation; weak voice; reduced pharyngeal reflex; cylindrical chest; unable to hold the head, roll over, sit, or stand; tendon reflexes are not elicited from the legs, and are significantly reduced from the arms.	administered by decision of the medical commission.	to the stomach, does not sit on her own, does not stand, and does not walk; the range of active movements is reduced, and the muscle strength in the arms is 3 points and in the legs is 2 points; the patient has a 2nd degree of scoliosis; there are no tendon reflexes in the upper and lower extremities, and no abdominal reflexes; the patient's pelvic functions are intact, and they do not have any toilet training; the patient's voice has become slightly stronger; the pharyngeal and palatal reflexes are reduced, and the patient has dysphagia; the patient has a well-modulated babbling voice and can produce individual syllables.	
**9804.1 [9]**	*SMN1* (NM_000344.4):	From the patient's medical history, it is known that she has had gait disturbances	Nusinersen (16 years)	Neurological examination at the age of 17: height of 167 cm, weight of 62 kg; left-sided thoracic-lumbar scoliosis of the	At the age of 16: HFMSE score of 21

# DNA	Genotype	Symptoms on the first visit	Gene-based therapy (age at onset)	Symptoms (age at last examination)	Assessment of motor development scales
	c.821C>T (p.Thr274Ile)<br>*SMN1* – 1 copy<br>*SMN2* – 2 copies	and difficulty climbing stairs since the age of 2.5. She has not walked since the age of 9.		second degree; full range of head movements; full range of shoulder movements; atrophy and fibrillation on the tongue; good head control, does not lift the head from a lying position; difficulty in turning to the right and left; sitting with a kyphotic back in the thoracic spine; sitting independently with the use of external support; cannot stand on all fours for a long time; does not walk; wing-shaped shoulder blades; flat-valgus feet; contractures in the ankle joints; distal hypotrophy; limited passive and active movements in the limbs; reduced muscle strength in the upper limbs to 3 points, tendon reflexes are not elicited; limited movement in the hip joints, full range of movement in the knee joints, limited movement in the ankle	points. At the age of 17: HFMSE score of 21 points (stable).

# DNA	Genotype	Symptoms on the first visit	Gene-based therapy (age at onset)	Symptoms (age at last examination)	Assessment of motor development scales
				joints; reduced muscle strength in the lower limbs to 2 points, tendon reflexes are not elicited.	
**8826.1 [9]**	*SMN1* (NM_000344.4): c.821C>T (p.Thr274Ile) *SMN1* – 1 copy *SMN2* – 1 copy	Early development: held his head from 3 months, turned over from 5 months, sat from 7 months, walked independently with support from 8.5 months, and spoke some words from 12 months. At the age of 10 months, the child began to walk less well with support (slower, and there was weakness in his legs). At 1 year and 3 months, he could only take a few steps with support, and then, over the next three months, there was a regression of motor skills: he stopped walking, and his muscle weakness increased. At 1 year and 4 months, he stopped standing on his feet, sitting independently, and turning over, and he sat with a weak back.	Nusinersen (7 years)	Neurological examination at the age of 10: height 134 cm, weight 20.3 kg; right-sided thoracic-lumbar scoliosis of degree IV; bilateral congenital hip dislocation, equinus foot deformity; full range of head movements, no difficulty in raising the shoulders; fasciculations, tongue muscle atrophy; holds the head confidently while sitting, and does not raise it from a lying position; turns to the side with difficulty, does not turn back to the back; does not sit on his own, sits briefly with a kyphotic back using his hands; does not walk, crawl, or stand on all fours; ulnar positioning of the hands; elbow joint contractures; upper limb muscle strength	At the age of 9: CHOP INTEND - 41 points.

# DNA	Genotype	Symptoms on the first visit	Gene-based therapy (age at onset)	Symptoms (age at last examination)	Assessment of motor development scales
				reduced to 2 points proximally, does not raise the arms above the horizontal level; diffuse reduced muscle tone, no tendon reflexes; limited movement in the hip joints, contractures in the knee and ankle joints; lower limb muscle strength reduced to 1 point proximally, 1-2 points distally, no tendon reflexes.	
**8118.1 [9]**	*SMN1* (NM_000344.4): c.835-18_835-15del<br>*SMN1* – 1 copy<br>*SMN2* – 1 copy	From the neurologist's examination at the age of 15 years and 4 months, the following is known: asymmetry of the shoulder blades, waist triangles, and spinal curvature; reduced range of active and passive movements in all limbs, especially in the lower extremities; contracture of the knee joints; equinovarus deformity of the feet; diffuse muscle hypotonia, muscle strength in the arms: 3 points in the distal regions, 0 points in the distal regions, 2 points in the	Risdiplam (17 years)	Neurological examination at the age of 17: height of 158 cm, weight of 52.5 kg; reduced muscle tone; left-sided lumbar scoliosis of the second degree; equino-ploskovalgus foot deformity; flexion contractures of the knee joints; intelligence at the lower limit; holds the head satisfactorily, compensates when leaning forward to the central position, and raises it from a lying position; turns to the right and left with difficulty; sits	At the age of 18: RULM scale - 30 points (+4 points per year), HFMSE - 20 points (stable).

# DNA	Genotype	Symptoms on the first visit	Gene-based therapy (age at onset)	Symptoms (age at last examination)	Assessment of motor development scales
		proximal regions, tendon reflexes: reduced D=S in the biceps and triceps, absent in the carpo-radial, knee, and Achilles reflexes; unable to walk independently; pelvic disorders in the form of urinary and fecal incontinence; pronounced hyperhidrosis of the palms and soles, cold feet that are red to purple during the examination. Mental status: available for contact, follows simple instructions, and has a slightly reduced intellect. Functional scale testing in May 2023: RULM - 26 points, HFMSE - 20 points.		with a kyphotic back in the thoracic and lumbar regions of the spine; does not sit on his own; stands on his own and with support; has a kyphotic posture with "winged scapulae"; equino-varus foot positions; contractures in the ankle joints; reduced muscle strength in the upper extremities: 4 points proximally, 3 points distally, reduced tendon reflexes in the hands; passive movements in the knee and ankle joints are limited; reduced muscle tone, reduced muscle strength in the lower extremities to 1-2 points proximally, 3 points distally; tendon reflexes are not elicited.	
**OD1**	*SMN1* (NM_000344.4): c.821C>T (p.Thr274Ile)	Early development: held his head from 1.5 months, turned over from 3.5 months, never sat up on his own, did not crawl, and did not support himself on his feet.	Nusinnersen (6 months) Onasemnogene abeparvovec	Against the background of treatment with Nusinersen, there is an increase in muscle strength and tone, the voice has become louder, and the patient can sit	No data

# DNA	Genotype	Symptoms on the first visit	Gene-based therapy (age at onset)	Symptoms (age at last examination)	Assessment of motor development scales
	SMN1 – 1 copy<br>*SMN2* – 1 copy		(12 months)	independently for a long time without support and manipulate toys. Examination at 12 months after administration of Onasemnogene abeparvovec: Psycho-speech development is age-appropriate, and the patient can fully rotate their head. There is no difficulty in raising their shoulders. The "free shoulder" symptom is positive, and there are fasciculations on the tongue. The patient holds their head satisfactorily, controls their head movements, and can lift their head when pulled by their arms. However, they cannot lift their head from a lying position on their stomach. When lying on their stomach, they can lift their head, and they can stand on their elbows while holding their head. The patient can turn	

# DNA	Genotype	Symptoms on the first visit	Gene-based therapy (age at onset)	Symptoms (age at last examination)	Assessment of motor development scales
				from the back to the stomach and back again. The patient cannot sit independently, but they can sit with kyphoscoliosis. They can manipulate objects while sitting, and they can also lift their arms above the horizontal level to eye level. The patient cannot stand, walk, or stand on all fours. There is no support for their legs. The patient has flat feet with valgus deformity. There are contractures in the knee joints. The upper extremities: Passive movements in all joints are fully possible, but there is a limitation in pronation in the forearm. Active movements are limited due to muscle weakness. The muscle tone is hypotonic. The muscle strength is reduced, with a score of 3 in the proximal and distal regions. There are no tendon	

# DNA	Genotype	Symptoms on the first visit	Gene-based therapy (age at onset)	Symptoms (age at last examination)	Assessment of motor development scales
				reflexes. The lower extremities: Movements are limited due to muscle weakness. The muscle tone is hypotonic. The muscle strength is reduced to 2-3 points. There are no tendon reflexes. The patient's sensitivity is approximately normal. The patient reaches for toys with intention, and there is tremor in their hands. There are no pyramidal symptoms.	
**6.3472**	*SMN1* (NM_000344.4): c.80A>C (p.Gln27Pro) *SMN1* – 1 copy *SMN2* – 1 copy	At the age of 18, weakness in the extremities appeared and increased, and the extremities became thinner. The disease had a progressive course. According to the results of an electroneuromyography (ENMG) and a pathohistological examination at the age of 28, spinal amyotrophy (proximal form, late onset) was more likely. At the age of 42, the patient had atony and atrophy of the muscles	Risdiplam (43 years)	Muscle strength has increased slightly, general well-being has improved, walking function has improved, and pain in the back and neck has been relieved, making it easier to turn over in bed. At the age of 43, the patient's muscle strength in the extremities was reduced (3 points in the distal arms, 2 points in the proximal arms, 3 points in the proximal	No data

# DNA	Genotype	Symptoms on the first visit	Gene-based therapy (age at onset)	Symptoms (age at last examination)	Assessment of motor development scales
		in the extremities, back, shoulder girdle, pectoral muscles, and thigh muscles, and reduced muscle strength in the extremities (3 points in the distal arms, 2 points in the proximal arms, 3 points in the proximal legs, and up to 1 point in the distal legs). The patient could not stand up from a sitting or lying position without assistance, and the tendon reflexes were very low.		legs, and up to 1 point in the distal legs), and he walked around the house using assistive devices. When standing up from a seated position, he extended his legs forward, and his tendon reflexes were very low.	
**6.4308**	*SMN1* (NM_000344.4): c.*3+1del<br>*SMN1* – 1 copy<br>*SMN2* – 2 copies	At the age of 2, she noticed a change in her gait and difficulty climbing stairs. At the age of 11, she stopped walking. At the age of 46, she was examined and found to have reduced neck muscle strength. She also had tetraparesis, with proximal movement in her arms (worse on the left side), flexion/extension of her forearms and hands, and a paresis score of 2.5-3 (worse on the left side). In her legs, she had proximal paresis	Risdiplam (47 years)	There is a clear positive trend in the form of increased muscle strength in the rectus muscles of the back, which is now capable of maintaining a sitting position on its own.	No data

# DNA	Genotype	Symptoms on the first visit	Gene-based therapy (age at onset)	Symptoms (age at last examination)	Assessment of motor development scales
		and limited distal movement. She also had contractures in her hip, knee, and ankle joints, as well as swelling in her legs. She had hyporeflexia and mild tremors in her hands.			
